# Protective Effect of PEG-EDTA and Its Zinc(II) Complex on Human Cells

**DOI:** 10.3390/ijms27010044

**Published:** 2025-12-20

**Authors:** Tashneet Dhaliwal, Cole Babcock, Brynmar Degenhardt, Isaac Osorio Passos, Tigran Stepanyan, Makan Golizeh

**Affiliations:** 1 Metals in Environment and Health (MEH) Research Cluster, Concordia University of Edmonton, Edmonton, AB T5B 4E4, Canada; 2 Department of Environmental and Physical Sciences, Faculty of Science, Concordia University of Edmonton, Edmonton, AB T5B 4E4, Canada; 3 Babcocks Synthetics Ltd., Wainwright, AB T9W 1PS, Canada; 4 Department of Biological Sciences, Faculty of Science, Concordia University of Edmonton, Edmonton, AB T5B 4E4, Canada; 5 School of Engineering, Faculty of Nanotechnology Engineering, Universidad Pontificia Bolivariana, Medellín 050031, Colombia

**Keywords:** chelation, cytotoxicity, EDTA, human umbilical vein endothelial cells (HUVECs), metal displacement, PEG-EDTA, PEGylation, spectroscopy, titration, triple-negative breast cancer (TNBC)

## Abstract

The most widely used chelating agent, ethylenediaminetetraacetic acid (EDTA), can cause mild to serious side effects when used for clinical applications. Introducing a polyethylene glycol (PEG) moiety into the molecular structure of EDTA has been shown to lower its toxicity; however, it is unclear whether this could affect EDTA chelation efficiency due to the steric hindrance and the loss of a coordination site caused by the PEGylation reaction. This research aimed to determine if PEGylation could reduce EDTA toxicity without affecting its chelation efficiency. To this end, effective formation constants were determined for EDTA and PEG-EDTA rare earth metal ion complexes using spectrophotometric and titrimetric methods. The stability of PEG-EDTA complexes with the target metal ions was assessed under different conditions using Fourier-transform infrared spectroscopy. The cytotoxicity and metal detoxification capacity of EDTA, PEG-EDTA, and their zinc(II) complexes were determined in two selected human cell types exposed to toxic heavy metal ions. This study suggests that PEG-EDTA has lower toxicity than EDTA, especially when complexed with a nontoxic metal ion, such as zinc(II), while only slightly losing chelation efficiency, potentially making PEG-EDTA a more favourable metal detoxification reagent for clinical applications.

## 1. Introduction

Ethylenediaminetetraacetic acid (EDTA) is a polycarboxylate chelating agent that strongly binds to metal ions via its nitrogen and oxygen atoms, forming a metal ion-ligand complex [1]. This powerful chelating agent has proved useful in various analytical, clinical, and commercial applications. EDTA is a synthetic compound found in many everyday items, such as food products and personal care items, acting as a preservative to inactivate metal ions, which can otherwise catalyze oxidative reactions and affect product stability [2]. EDTA has also been commonly used in chelation therapy, a medical procedure in which a chelating agent is administered to an individual with heavy metal poisoning to scavenge toxic metal ions, such as lead(II) and mercury(II) [3]. EDTA is typically delivered to the patient via intramuscular injection or intravenous infusion, as it is absorbed poorly in the gastrointestinal tract, and can only effectively disperse via the circulatory system [4]. Heavy metal exposure directly impacts morbidity by influencing cell death through increased oxidation and inhibition of vital metabolic processes; hence, systemic removal of heavy metal ions in an efficient manner is important [2]. Although EDTA can efficiently remove toxic metal ions from patients, it can also cause harmful side effects, such as the depletion of biologically significant metal ions (e.g., calcium, copper, iron, and zinc ions) essential to cellular processes (e.g., enzymatic reactions), causing arrhythmias, hypocalcaemia, renal toxicity, and tetany [5,6]. These adverse effects have limited the use of EDTA for heavy metal detoxification. Therefore, modifying the chemical structure of EDTA to alleviate some of these side effects would be of clinical interest.

PEGylation is a chemical modification technique by which polyethylene glycol (PEG) is covalently integrated into the molecular structure of a target compound primarily to alter its chemical and physical properties [7]. These alterations may include enhanced solubility, improved stability, and increased biocompatibility to boost the overall efficacy of the modified compound in biological systems [7,8]. Previous studies have shown that PEGylation can also decrease the toxicity of the modified compounds. For example, PEGylated polyethyleneimine and PEGylated wortmannin were less cytotoxic than their unmodified forms despite maintaining their biological properties [9,10]. However, it is unclear whether PEGylation could decrease EDTA toxicity without affecting its ability to chelate heavy metal ions. In theory, covalent addition of a bulky functional group, such as a PEG moiety, to the ligation sites of the EDTA molecule could increase steric hindrance around those sites and potentially interfere with the complexation reaction. A previous study has shown that PEGylation can decrease the binding efficiency of certain biomolecules towards their targets [11]. Another study demonstrated that PEGylation hindered the binding of a therapeutic compound to a bacterial membrane, reducing its antibacterial activity [12]. Further, PEGylated enzymes have been found to have lower binding affinities to their peptide substrates because of the added steric hindrance from the PEG moiety [13].

The overarching goal of this study was to determine if PEGylation altered the metal ion-ligation ability of EDTA or its toxicity in humans. The chelation efficiency and metal ion-complex stability of PEGylated EDTA (PEG-EDTA) were determined and compared to those of unmodified EDTA using terbium(III) and zirconium(IV) as target heavy metal ions. Quantitative assessment of metal ion chelation efficiency was performed by spectrophotometric and titrimetric methods to selectively quantify the free metal ions in the presence of chelating agents. Fourier-transform infrared (FT-IR) spectroscopy was used to evaluate the stability of metal ion-chelating agent complexes under various conditions, such as pH, salinity, and UV exposure. Finally, the cytotoxicity of EDTA, PEG-EDTA, and their zinc(II) complexes was compared using two different human cell models.

## 2. Results

### 2.1. Chelation Efficiency of PEG-EDTA

EDTA and PEG-EDTA chelation efficiencies were calculated for two selected rare earth metal ions, Tb(III) and Zr(IV), as percent chelated metal ion in solution using complexation colorimetry or displacement titration methods. Both methods generated reproducible results; however, the % chelation efficiency obtained for each chelation reaction varied (*p* < 0.01) between the methods. Both methods revealed a statistically significant difference (*p* < 0.05) between EDTA and PEG-EDTA chelation efficiencies, with PEG-EDTA being 1.5–8.0% less efficient than EDTA. The chelation efficiencies of both chelating agents were greater (*p* < 0.01) when binding with Zr(IV) than with Tb(III). Table 1 summarizes the % chelation efficiencies obtained for EDTA or PEG-EDTA with Tb(III) or Zr(IV) ions using the colorimetry or titration method.

### 2.2. Physicochemical Properties of PEG-EDTA and Its Metal Ion Complexes

The solubility of PEG-EDTA in 25% NaCl was tested and compared to that of EDTA under three conditions: gentle shaking (a few seconds), vigorous mixing (30 s), and heated incubation (80 °C for 20 min). While EDTA was only soluble in brine when heated, PEG-EDTA dissolved almost immediately under every solubilization condition. Both Tb(PEG-EDTA) and Zr(PEG-EDTA) were also readily soluble in brine, up to 1.0 M NaCl, as well as all other tested buffers. The PEG-EDTA synthesized for this study was an amber oily material, and its Tb(III), Zn(II), and Zr(IV) complexes were amber transparent solids.

**Scheme 1:**
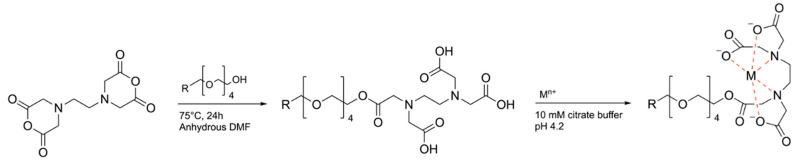
Chemical synthesis of propargyl-PEG_4_-EDTA and its metal ion complexes. DMF: dimethylformamide; M^n+^: metal ion; R: propargyl. Dashed lines signify metal ion coordination.

The effect of pH, salinity (molarity of NaCl in solution), temperature, and UV light exposure on PEG-EDTA and its Tb(III) and Zr(IV) complexes was also tested. Two stretching frequencies were used as a surrogate to assess the stability of PEG-EDTA and its metal ion complexes under the test conditions: the PEG-EDTA ester C=O bond, and the carboxyl C=O bond adjacent to the chelation site (see Scheme 1). The stretching frequency of the ester C=O (${\bar{\nu }}_{e}$) varied between 1731.00 ± 0.21 cm^−1^ for uncomplexed PEG-EDTA and 1747.79 ± 0.12 cm^−1^ for Zr(PEG-EDTA) in 1.0 M NaCl. The stretching frequency of the carboxyl C=O (${\bar{\nu }}_{c}$) changed between 1590.96 ± 0.04 cm^−1^ for Tb(PEG-EDTA) at pH 10.0, and 1668.59 ± 0.06 cm^−1^ for unmodified EDTA trisodium salt.

Overall, both Tb(III) and Zr(IV) complexes had significantly higher ${\bar{\nu }}_{e}$ values than uncomplexed PEG-EDTA (ANOVA *p* = 2.25 × 10^−21^ and 1.53 × 10^−15^ for Tb(III) and Zr(IV), respectively). The Zr(PEG-EDTA) complex had significantly lower ${\bar{\nu }}_{e}$ values at higher pH, decreasing from 1745.84 ± 0.16 cm^−1^ at pH 2.2 to 1735.95 ± 0.18 cm^−1^ at pH 10.0. Tb(PEG-EDTA) showed a significant but opposite trend, i.e., the frequencies increased from 1732.67 ± 1.85 cm^−1^ at pH 2.2 to 1741.58 ± 0.08 cm^−1^ at pH 10.0. Salinity (represented by mol/L NaCl) had a significant effect on the ${\bar{\nu }}_{e}$ value of PEG-EDTA with both metal ions. An increased concentration of NaCl increased this frequency in PEG-EDTA complexes with Tb(III) and Zr(IV). Higher temperatures caused the ${\bar{\nu }}_{e}$ of Tb(PEG-EDTA) and Zr(PEG-EDTA) to decrease; however, only the change in the ${\bar{\nu }}_{e}$ of the Tb(III) complex was significant. UV light (365 nm) exposure had no significant effect on the ${\bar{\nu }}_{e}$ of Tb(PEG-EDTA). However, UV exposure caused ${\bar{\nu }}_{e}$ to significantly increase in Zr(PEG-EDTA) after 5 min.

Tb(III) and Zr(IV) complexes with PEG-EDTA had significantly lower ${\bar{\nu }}_{c}$ values than uncomplexed PEG-EDTA (ANOVA *p* = 6.95 × 10^−34^ and 1.20 × 10^−13^ for Tb(III) and Zr(IV), respectively). While Tb(PEG-EDTA) had significantly lower ${\bar{\nu }}_{c}$ values at higher pH, the ${\bar{\nu }}_{c}$ of Zr(PEG-EDTA) was unaffected by pH. Similarly, NaCl molarity, temperature, and UV exposure significantly increased the ${\bar{\nu }}_{c}$ of the PEG-EDTA complex with Tb(III) but did not have any significant effect on that of Zr(IV).

The FT-IR measurements were highly reproducible with coefficients of variation between 0 and 0.2%. Table 2 summarizes FT-IR stretching frequencies of the ester C=O and carboxyl C=O bonds in PEG-EDTA complexes with Tb(III) and Zr(IV) ions under various pH, salinity, and UV exposure conditions.

### 2.3. Cytotoxicity and Metal Detoxification Ability of PEG-EDTA

Two groups of human cells were treated under physiological conditions with viable concentrations of EDTA or PEG-EDTA and two cytotoxic heavy metal ions. Human umbilical vein endothelial cells (HUVECs) (1.6 × 10^5^ cells/replicate) were treated with lethal concentrations of Pb(II), based on a previous report [14], with or without the chelating agent. Triple-negative breast cancer (TNBC) cells (7.4 × 10^5^ cells/replicate) were exposed to chelated Ag(I). Negative (untreated), positive (metal ion-only), and chelating agent-only controls were included in both experiments.

Both HUVECs and TNBC cells were >95% viable in the absence of heavy metal ions or the chelating agents. Pb(II) treatment decreased the viability of HUVECs to 42.3 ± 3.6% (*p* = 0.002, with respect to the negative control). Both EDTA and PEG-EDTA enhanced the viability of HUVECs in the presence of Pb(II) (*p* = 0.03 and *p* = 0.06 for EDTA and PEG-EDTA, respectively, with respect to the positive control). However, neither group was as viable as the untreated control. Pre-complexing the chelating agents with Zn(II), i.e., adding Zn(EDTA) or Zn(PEG-EDTA) to the cells instead of EDTA or PEG-EDTA, further increased the viability of Pb(II)-treated HUVECs (*p* > 0.05, both, with respect to uncomplexed chelating agents; *p* = 0.01 and *p* = 0.2 for EDTA and PEG-EDTA, respectively, with respect to the positive control). Pb(II)-treated HUVECs given Zn(II)-loaded PEG-EDTA had the closest viability to the negative control (*p* = 0.05).

**Figure 1:**
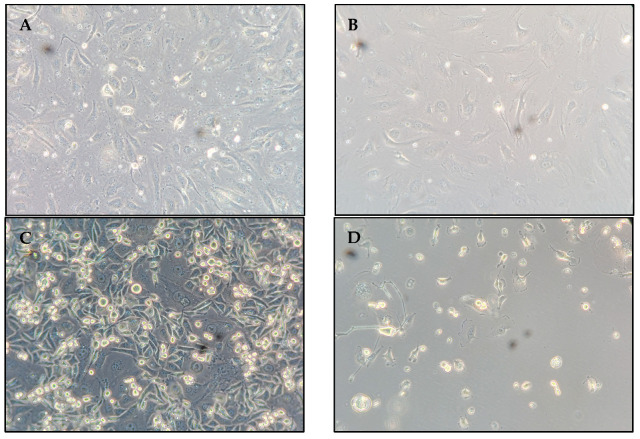
Microscopy images from untreated HUVECs (**A**), Pb(II)-treated HUVECs (**B**), untreated TNBC cells (**C**), and Ag(I)-treated TNBC cells (**D**) under 10× magnification after 24 h of incubation with treatment media.

The TNBC cells exposed to Ag(I) were only 13.5 ± 0.3% viable (*p* = 0.002, with respect to the negative control). Both EDTA and PEG-EDTA significantly increased the viability of the cells in the presence of Ag(I) (*p* = 0.01 and *p* = 0.003 for EDTA and PEG-EDTA, respectively, with respect to the positive control). Cells treated with EDTA or PEG-EDTA alone were nearly as viable as the negative control (*p* > 0.05, both). See Table 3 for the cell viability data of the HUVEC/Pb(II) and TNBC/Ag(I) experiments. Figure 1 exhibits the untreated and treated cells under the microscope.

## 3. Discussion

In this study, two methods were developed to determine the chelation efficiency of PEGylated and unmodified EDTA with selected heavy metal ions. This information was used to assess the impact of PEGylation on the efficiency of EDTA as a chelating agent to bind heavy metal ions in aqueous systems. The solubility and stability of selected PEG-EDTA metal ion complexes were later evaluated under different conditions using an FT-IR spectroscopy-based approach. Furthermore, the utility of PEG-EDTA as a heavy metal ion chelating agent was tested on two human cell types exposed to toxic heavy metal ions, and it was compared to that of the unmodified EDTA.

Our findings demonstrated that both analytical methods developed in this study were reproducible, with relative standard deviations varying in the range of 0.002–0.006% for the colorimetric assay, and 0.2–1.0% for displacement titration. This is consistent with spectrophotometric measurements being intrinsically more reproducible than titrimetric analyses. The results obtained from these two methods were, however, significantly different, with the titrimetric method showing a greater chelation efficiency for EDTA and PEG-EDTA than the colorimetric analysis (ANOVA *p* = 0.0002). Inductively coupled plasma mass spectrometry (ICP-MS) analysis of another sample set (data not included) confirmed that the colorimetric method was more accurate than the titration. This could be due to the titration error caused by the difficulties in discerning the colour change, i.e., light pink to pale yellow, at the endpoint of the titration. 

The data provided through spectrophotometric and titrimetric analyses suggest that PEGylation of EDTA can decrease its chelation efficiency, as seen with Tb(III) and Zr(IV) ions. Our chelation assessment showed that Tb(III) complexes were 1.5–2.3% less stable with PEG-EDTA than with EDTA (*p* < 0.01, both methods). Likewise, Zr(IV) complexes were 3.9–8.0% less stable with PEG-EDTA than with EDTA (*p* < 0.001 colorimetry; *p* < 0.05 titration). The difference in chelation efficiency between Tb(III) and Zr(IV) was also consistent with their formation constants (log K_f_) found in the literature, with EDTA having a greater affinity towards Zr(IV) over Tb(III) [15].

Tb(III) and Zr(IV) form chelation products that are among the most stable EDTA complexes (log K_f_ = 17.9 and 29.3, respectively). These ions have thus been selected in place of more naturally abundant metal ions, such as Ca(II) and Fe(II) (log K_f_ = 10.6 and 14.3, respectively) to ensure a sensitive comparison of the chelation efficiencies of EDTA and PEG-EDTA.

The decreased coordination sites and increased steric hindrance caused by the addition of the PEG moiety may account for the observed decrease in binding efficiency of EDTA when PEGylated. EDTA typically binds to metal ions through its two nitrogen atoms, as well as the four oxygen atoms in each carboxyl head [1]. When EDTA is PEGylated, one carboxyl group is lost to form the PEG ester (see Scheme 1). Losing one metal ion-binding site is likely to cause a decrease in the binding efficiency, as the hexadentate EDTA becomes a pentadentate chelating agent instead. This can also cause a decrease in the total electrostatic interaction between EDTA and the metal ion, forming a less stable EDTA-metal ion complex. Moreover, a ligand that can form a complex at more than one binding site can further stabilize the complex through the chelate effect, in which the affinity of the ligand toward the metal ion is increased due to the formation of a 3D structure that surrounds the metal ion [16,17]. Losing a binding site could, therefore, decrease complex stability due to a weaker chelate effect. An increase in steric hindrance within EDTA may also lead to weaker binding of the chelating agent with the metal ion, thus forming a less stable complex [18]. These can potentially explain why PEG-EDTA demonstrated a decrease in chelation efficiency in comparison to unmodified EDTA.

The results of the complex stability analyses indicate that PEG-EDTA can efficiently bind to metal ions within a broad pH range, granting it potential as a candidate chelating agent for clinical applications [19]. The increased solubility of PEG-EDTA grants more weight to the assertion of its candidacy. Once the PEG-EDTA-metal ion complex is formed, the higher solubility may allow it to be excreted at a higher rate through the renal system, as higher solubility compounds are generally absorbed by the kidneys and excreted more readily [20]. The stability analysis also showed that PEG-EDTA and its metal ion complexes are relatively resistant to heat, salinity, and UV exposure. Considering its higher solubility than EDTA, PEG-EDTA could be a more ideal candidate for environmental monitoring and remediation applications, such as detection and removal of heavy metals in wastewater [21]. Moreover, the increased water solubility of PEG-EDTA makes it more bioavailable than its non-PEGylated counterpart. However, while EDTA and its transition metal ion complexes are typically crystalline solids, PEG-EDTA was an oil, and although its tested heavy metal ion complexes were solid materials, this can limit the practicality of PEG-EDTA in clinical or environmental applications, for example, when accurate weighing or transferring is required. It is noteworthy that the PEG-EDTA used in this study includes an alkynyl moiety, which enables covalent linkage via click chemistry for applications such as bioconjugation or surface immobilization. However, the inclusion of an alkyne may affect the compound’s physicochemical properties.

Our data suggest that PEG-EDTA can protect human cells against heavy metal-induced toxicity. Both EDTA and PEG-EDTA were able to protect HUVECs from lead(II) poisoning; however, EDTA was more efficient (77.4 ± 7.7% cell viability vs. 71.0 ± 12.1% for PEG-EDTA; *p* > 0.05). This is consistent with PEG-EDTA having a lower chelation efficiency than EDTA, as described earlier. Similarly, while TNBC cells had great mortalities in the presence of silver(I) (13.5 ± 0.3% cell viability), their survival rates significantly increased when treated with EDTA or PEG-EDTA (*p* = 0.01 and *p* = 0.003, respectively); however, PEG-EDTA was a more effective heavy metal detoxification agent than EDTA (*p* > 0.05). While the underlying cause of this discrepancy is unclear, previous research has shown that EDTA analogues and derivatives, such as 1,2-bis(2-aminophenoxy)ethane-N,N,N′,N′-tetraacetic acid (BAPTA) [22], and diethylenetriaminepentaacetic acid (DTPA) [23], have lower anticancer properties than EDTA, i.e., improve cancer cell viability.

HUVECs are sensitive to oxidative stress [24]. Notably, prooxidant heavy metal ions, such as lead(II), promote oxidative stress by catalyzing the formation of reactive oxygen species [25]. Therefore, we have selected HUVECs to ensure a sensitive response to lead(II)-induced toxicity. Conversely, TNBC cells are known to be resilient to chemically induced stress [26]. However, they are extremely susceptible to silver(I) ions [27]. These cell types have been selected to demonstrate the protective effect of PEG-EDTA against heavy metal ions that are otherwise toxic towards their corresponding cells. Furthermore, lead(II) is among the most common sources of heavy metal poisoning in humans [28]. Assessing the detoxification capacity of PEG-EDTA on this ion would thus be of clinical importance. Silver(I) poisoning is less common. However, silver(I) ions and nanoparticles are increasingly incorporated into commercial products, such as cosmetics and textiles. Recent studies suggest that both silver(I) ions and nanoparticles are toxic to humans [29].

A major problem with EDTA Is the depletion of essential metal Ions, such as calcium and zinc ions, from the cell (or in clinical applications, the patient) [30]. Pre-complexing EDTA to a low-affinity essential metal ion, such as Ca(II), could alleviate this problem. Previous studies have shown that calcium disodium edetate (CaNa_2_EDTA) is a better treatment for toxic metal poisoning than uncomplexed EDTA, as toxic metal ions, such as Pb(II), can displace Ca(II) in the complex [31]. This is because most heavy metal ions generally have a higher affinity for EDTA than Ca(II). For example, Pb(II) (log K_f_ = 18.0) can readily displace Ca(II) (log K_f_ = 10.6) in an EDTA complex, while the excess EDTA remains complexed with Ca(II), reducing its ability to deplete other essential metal ions present in the body. A similar approach was employed in this study, although using Zn(II) instead of Ca(II). Zn(II) has a larger affinity for EDTA (log K_f_ = 16.5) than most other metal ions that are essential for the cell, such as Fe(II) (log K_f_ = 14.3) and Mn(II) (log K_f_ = 13.9), thus preventing their excessive depletion. Previous studies have suggested that Zn(II) may have protective effects against heavy metal- and metalloid-induced toxicity [32,33]. Our study suggests that Zn(II)-complexed EDTA or PEG-EDTA was more effective in protecting HUVECs than their uncomplexed counterparts, with Zn(PEG-EDTA) being 6.5 ± 7.2% more effective (in terms of cell viability) than Zn(EDTA), although the difference was not significant. We postulate that the toxic Pb(II) ions have displaced the non-toxic Zn(II) ions in Zn(EDTA) or Zn(PEG-EDTA) complexes due to Pb(II) ions’ larger affinity for the chelating agents [log K_f_ for EDTA = 18.0 for Pb(II) vs. 16.5 for Zn(II)], allowing Pb(II) ions to be safely removed from the system via the following reaction: Pb^2+^ + Zn(EDTA) → Zn^2+^ + Pb(EDTA). PEGylation has been shown to lower chemical toxicity in biological environments [9,10]. For example, PEGylated polyamidoamine dendrimers exhibited less cytotoxicity than their unmodified version, potentially due to the steric hindrance caused by PEGylation [34]. The charge shielding ability of PEG can also increase biocompatibility, reducing cytotoxicity in the target [35]. We did not observe any significant loss of cell viability when TNBC cells were treated with EDTA or PEG-EDTA (92.6 ± 6.2% and 92.4 ± 2.9% cell viability for EDTA and PEG-EDTA, respectively; *p* > 0.05, both), suggesting neither chelating agent was cytotoxic. However, the HUVEC experiment postulates that Zn(PEG-EDTA) was more biocompatible than uncomplexed PEG-EDTA [89.6 ± 4.2% cell viability for Zn(PEG-EDTA) vs. 71.0 ± 12.1% for PEG-EDTA], although the difference was not significant.

## 4. Materials and Methods

### 4.1. Materials and Equipment

Propargyl-PEG_4_-alcohol was purchased from Broad Pharm (San Diego, CA, USA). HUVECs (ATCC Cat. # CRL-1730), MDA-MB-231 TNBC cells (ATCC Cat. # HTB-26), F-12K cell culture medium (ATCC Cat. # 30-2004), and trypsin/EDTA (ATCC Cat. # 30-2101) were purchased from Cedarlane Labs (Burlington, ON, Canada). Heparin sodium salt (Cat. # H3393-10kU) and endothelial cell growth supplement (ECGS) (Cat. # E2759) were obtained from Sigma-Aldrich Canada (Oakville, ON, Canada). Premium-grade Gibco Fetal Bovine Serum (FBS) (Cat. # A5670401) was purchased from Fisher Scientific (Hampton, NH, USA). All other reagents were purchased from Fisher Scientific or Sigma-Aldrich Canada.

Dimethylformamide was dried over prepared 4-angstrom molecular sieves, under a nitrogen atmosphere, for three days before use. All other reagents were used directly as obtained, without further purification or alteration. The Ag(I), Pb(II), Tb(III), Zn(II), and Zr(IV) aqueous ions were produced from silver(I) nitrate, lead(II) acetate trihydrate, terbium(III) chloride hexahydrate, zinc(II) nitrate hexahydrate, and zirconium(IV) chloride (anhydrous) adducts, respectively. To minimize monovalent and divalent metal interferences, all experiments, except titrimetric measurements, were conducted in plastic labware, when possible. Unless otherwise specified, the solvent was ultrapure water (18.2 MΩ·cm at 25 °C) obtained from a Millipore (Burlington, MA, USA) Milli-Q IQ 7000 water system.

Thin-layer chromatography was performed using TLC silica gel 60 F_254_ aluminum sheets (EDM Millipore, Bulington, MA, USA). FT-IR analysis was conducted using a PerkinElmer (Waltham, MA, USA) Spectrum Two spectrometer equipped with an attenuated total reflectance (ATR) sampling module. UV-visible spectrophotometry was performed in the visible region (colorimetry) with a single-beam PerkinElmer Lambda XLS+ spectrometer in 0.1 cm-wide quartz cuvettes.

High-resolution mass spectrometry (HR-MS) was performed at the University of Alberta Mass Spectrometry Facility using flow injection and an Agilent Technologies (Santa Clara, CA, USA) 6220 electrospray ionization-orthogonal acceleration time-of-flight (ESI-oaTOF) instrument, equipped with a dual-sprayer electrospray ionization source with the second sprayer providing a reference mass solution, and an Agilent 1200 Series isocratic pump. Optima LC-MS-grade methanol was used as a carrier solvent at a flow rate of 0.200 mL/min. Mass spectra were acquired in negative ionization mode, with a capillary voltage of 3500 V and a fragmentor voltage of −100 V. Drying gas was 10 L/min, and nebulizer pressure was 25 psi. 

### 4.2. PEG-EDTA Synthesis

Propargyl-PEG_4_-alcohol (1.0 g) and EDTA dianhydride (1.25 molar equivalent) were added to a 100 mL flame-dried, three-necked round-bottom flask with a condenser, thermometer, and stir bar under nitrogen gas. Anhydrous dimethylformamide (40.0 mL) was added, with stirring, to the flask via syringe. The mixture was then heated to 75 °C and held for 24 h. Reaction progress was monitored by thin-layer chromatography, using 1:4:1 acetic acid/n-butanol/water as the eluent, with potassium permanganate staining. Over the course of the reaction, the solution slowly turned from cloudy to clear, then to amber. Once the reaction was deemed complete, it was let cool to room temperature, and the bulk of the solvent was removed under vacuum and moderate heating using a rotary evaporator. Ultrapure water (40.0 mL) was added to the remaining viscous, amber liquid to precipitate the unreacted dianhydride. After the precipitate formed, the solution was evenly decanted into 15 mL plastic conical tubes and centrifuged at 4500× *g* for 20 min at room temperature. The supernatant was collected, transferred into a pre-weighed 100 mL pear-shaped flask, and lyophilized to yield the final product. The removal of the reactant was confirmed by FT-IR, i.e., the disappearance of the EDTA dianhydride carbonyl stretching peak at 1807 cm^−1^. The purity and molecular structure of the product was confirmed by FT-IR (1731 cm^−1^, C=O stretching; 2112 cm^−1^, C≡C stretching) and HR-MS (*m*/*z* 461.1777 for PEG-EDTA). Small amounts of disubstituted product, (PEG)_2_EDTA, was also identified (*m*/*z* 631.2737). No other side products or remaining reactants were identified. See FT-IR and HR-MS spectra in Supplementary Materials (Figures S1 and S2). The product was stored at −20 °C until used, without further purification.

### 4.3. Metal Ion-Chelating Agent Complexation

To prepare the metal ion-chelating agent complexes, a 3.00 mM EDTA or PEG-EDTA solution in ultrapure water was incubated in a 15 mL plastic tube with an equivalent volume of a 3.00 mM metal ion solution in 0.100 M acetate buffer pH 4.2. The solutions were heated to 80 °C for at least an hour, or until the solutions were clear. Metal ion-chelating agent solutions were then used for downstream analysis without further purification. The free (unreacted with EDTA or PEG-EDTA) metal ions were quantified using complexation colorimetry or nickel(II) displacement titration. The metal ions quantified by these methods include terbium(III) and zirconium(IV).

Scheme 1 exhibits the chemical reaction for the synthesis of propargyl-PEG_4_-EDTA, referred to in this article as PEG-EDTA, its reaction with metal ions, and the structure of the metal ion-chelating agent complex.

### 4.4. Quantitation of Free Metal Ions Using Complexation Colorimetry

Metal ion quantitation was performed using chrome azurol S (CAS) as the complexation reagent based on a published method [36]. Five metal ion-CAS standard solutions were prepared at the 0.0100 mM, 0.0250 mM, 0.0500 mM, 0.0750 mM, and 0.100 mM concentrations. To prepare the solutions in a 1:1 stoichiometric ratio, appropriate volumes of 1.00 mM CAS and 1.00 mM metal ion were mixed, diluted to 10.00 mL with ultrapure water, and incubated on a shaker (85 rpm) for 15 min to allow metal ion-CAS complexes to form.

**Figure 2:**
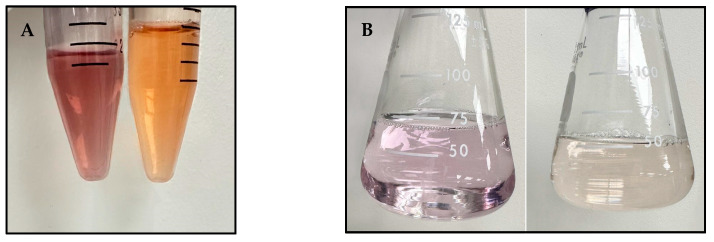
(**A**) The colour of Tb(III)-CAS (left) and Zr(IV)-CAS (right) complexes measured at 500 nm and 598 nm, respectively, used for spectrophotometric determination of metal ion chelation efficiencies; (**B**) Initial colour of the Cu-PAN indicator in presence of Ni(II) (left) and endpoint colour of Cu-PAN (right) used in displacement titrimetric determination of metal ion chelation efficiencies.

An equal volume of a 5.00 mM CAS solution was added to freshly prepared metal ion-chelating agent solutions and incubated on a shaker (85 rpm) for 15 min for metal ions to transfer to CAS. The colour change was used to determine the completion of the metal ion transfer reaction. Tb(III)-CAS complex formed an orange colour, while Zr(IV)-CAS was deep purple with a hint of red (Figure 2A). The absorbance was measured for both samples and standard solutions at either 500 nm (Tb) or 598 nm (Zr). Method blanks consisting of the 0.100 M acetate buffer pH 4.2, were included in each sample or standard measurement. Complexed and free metal ion concentrations were calculated using the external calibration method with least-squares regression for each metal ion-CAS complex. The chemical reactions for colorimetric determination of free Tb(III) and Zr(IV) with CAS complexation colorimetry are listed in Table 4 (reactions **A** and **B**, respectively). The analyses were performed in triplicate.

### 4.5. Quantitation of Free Metal Ions Using Displacement Titration

To 20.00 mL of the metal ion-chelating agent (sample) solution, 0.100 M acetate buffer pH 4.2 (3.00 mL), 5.00 mM tetracyanonickelate(II) solution (20.00 mL), and ultrapure water (17.00 mL) were added, and the resulting solution was incubated at 70–80 °C for 15 min. 5.00 mL of this solution was transferred into an Erlenmeyer flask along with 5.00 mL of 0.100 M acetate buffer at pH 4.2. Two drops of Triton X-100 were added, and then the solution was heated to 80 °C. The solution became cloudy when the surfactant dissolved. Following this, two drops of 0.01% copper 1-(2-pyridylazo)-2-naphthol (Cu-PAN) indicator were added to the sample solutions. Method blanks were composed of 0.100 M acetate buffer pH 4.2 (5.00 mL), Triton X-100 (two drops), and 0.01% Cu-PAN indicator (one drop).

A 0.500 mM EDTA solution was prepared as the titrant and standardized with a 1.00 mM calcium carbonate primary standard solution, with calmagite as the titration endpoint indicator. The sample was then titrated with the standardized EDTA solution at 80 °C. The endpoint was reached once the light pink solution turned pale yellow (Figure 2B). The Ni(II) displacement titration reactions for Tb(III) and Zr(IV) are listed in Table 4 (reactions **C** and **D**, respectively). The analyses were performed in triplicate.

### 4.6. Metal Ion Chelation Efficiency Determination

Chelation efficiencies were determined based on molar concentrations of free Tb(III) or Zr(IV) ions that instead of reacting with EDTA or PEG-EDTA reacted with CAS or displaced Ni(II) from the tetracyanonickelate(II) ions.

To calculate chelation efficiency for the selected metal ions, the following formula was employed regardless of the method used for complexed and free (uncomplexed) metal ion quantitation. The % chelation efficiency represents the amount of metal ion ($M^{n+}$) that reacted with the chelating agent and has been used as a surrogate for effective formation constants.

(1) $$\%\;Chelation\;Efficiency=\frac{\left[M_{total}^{n+}\right]-\left[M_{free}^{n+}\right]}{\left[M_{total}^{n+}\right]}\times 100\%$$

### 4.7. FT-IR Spectroscopy

To prepare the samples for ATR-FT-IR analysis, equal volumes of PEG-EDTA and metal ion solutions (at a 1:1 molar ratio) were incubated for 60 min in a 10 mM citrate buffer pH 4.0, and subsequently lyophilized. The PEG-EDTA metal ion complexes were exposed to different pH, salinity, temperature, and UV-exposure conditions. The pH tests were conducted by adding a pH-adjusted buffer (1.00 mL) to the lyophilized metal ion-chelating agent complex (25.0 mg) and gently shaking the mixture at room temperature for 90 min. The buffers used for this experiment were 10.0 mM citrate buffer pH 2.2, 10.0 mM phosphate buffer pH 6.5, 10.0 mM borate buffer pH 8.5, and 10.0 mM borate buffer pH 10.0. Salinity testing was performed by adding an NaCl solution (1.00 mL) to the lyophilized metal ion-chelating agent complex (25.0 mg) and gently shaking the mixture for 90 min. The NaCl solutions used in this test were at 0.0100 M, 0.100 M, and 1.00 M NaCl. To assess the effect of temperature on PEG-EDTA metal ion complexes, the complexes were incubated at 4, 60, and 90 °C in phosphate-buffered saline (PBS) pH 7.4. As for UV exposure, the lyophilized metal ion-chelating agent complex (25.0 mg) was exposed to UV light of a wavelength of 365 nm for 0.5, 1.0, and 5.0 min. The samples were re-lyophilized prior to ATR-FT-IR analysis. A no-metal ion control (i.e., uncomplexed PEG-EDTA) and an unmodified (i.e., non-PEGylated) control (EDTA trisodium salt) were also included in the experiment. All experiments were carried out in triplicate.

### 4.8. Solubility Testing

To assess the solubility of the chelating agents in various solvents, 5.00 mM solutions of EDTA and PEG-EDTA were prepared in a 25% *w*/*v* NaCl solution (1.00 mL). The solutions were observed for their solubility upon initial addition of the NaCl solution, after a few gentle shakes, and after vigorous mixing for 30 s. The samples were then heated to 80 °C in a heating block for 20 min. The experiment was performed in triplicate.

### 4.9. Human Cell Growth and Viability Testing

HUVECs were cultured in 10% FBS in F-12K media (i.e., Kaighn’s modification of Ham’s media) as a base media with heparin salt added at 0.1 mg/mL, and ECGS added at 0.03 mg/mL. The cells were seeded on two sterile polystyrene 6-well plates and incubated at 37 °C under 5% CO_2_ with complete media changes every 2–3 days. Regular passages were performed based on an assessment of confluence. With a confluence of 60%, the media was removed and replaced with the treated media of the respective groups.

MDA-MB-231 cells were cultured in 10% FBS in F-12K media and incubated at 37 °C under 5% CO_2_. The cells were grown to a 50% observed confluence before treatment. Treatment conditions were added to the growth media and filter-sterilized before a complete media change was performed using the newly prepared solutions.

The cultures were exposed to the treatment conditions overnight before a viability test was performed using a trypan blue staining procedure. The media was removed, and cells were detached using 0.25% trypsin-EDTA. Once the cells were observed to be detached, they were pelleted (5 min, 120× *g*) and resuspended in fresh untreated growth media. This cell solution was combined 1:1 (*v*/*v*) with trypan blue (10.00 µL cell solution to 10.00 µL of stain). Two solutions were made for each treatment, then counted on a hemocytometer, where the living cells counted were compared to the total cell count to obtain the cell viability for each treatment condition. Figure 1 exhibits microscopy images of selected HUVEC and TNBC cell samples.

### 4.10. Chemical Treatment of Human Cells

The toxicity of PEG-EDTA was tested on two types of human cells with and without a cytotoxic metal ion under physiological conditions. Unmodified EDTA was used as a comparator. The two cell types tested were normal/healthy HUVECs and TNBC (cancerous) MDA-MB-231 cells, exposed to Pb(II) and Ag(I), respectively.

Six different groups of HUVECs were included in the Pb(II)-induced toxicity assay. Two groups were treated with Pb(II) and either EDTA or PEG-EDTA to assess the effect of toxic metal exposure on the cells with or without metal ion chelation. Two other groups were treated with Pb(II) and either Zn(EDTA) or Zn(PEG-EDTA). The latter reagents were prepared by the 1:1 (mol/mol) reaction between Zn(II) and the chelating agent. These groups were added to assess the effect of metal ion displacement [antioxidant Zn(II) vs. prooxidant Pb(II)] on the cells. A positive control [containing Pb(II) but no chelating agent] and a negative control [containing no Pb(II) and no chelating agent] were also included in the experiment. The final concentration of Pb(II) and chelating agent in the culture media were 93 µM and 130 µM, respectively. The final concentration of Zn(II) was, therefore, equal to that of the chelating agent, i.e., 130 µM. All cell groups were incubated overnight at 37 °C under 5% CO_2_. Treatment conditions were assessed in triplicate.

Six different groups of MDA-MB-231 cells were included in the Ag(I)-induced toxicity assay. Two groups were treated with Ag(I) and either EDTA or PEG-EDTA to assess the effect of toxic metal exposure on the cells with or without metal ion chelation. Two groups were treated only with EDTA or PEG-EDTA in the absence of Ag(I) to assess the effect of the chelating agents on cell survival. A positive control [containing Ag(I) but no chelating agent] and a negative control [containing no Ag(I) and no chelating agent] were also included in the experiment. The final concentration of Ag(I) and the chelating agent in the culture media were both 176 µM. All cell groups were incubated overnight at 37 °C under 5% CO_2_. Treatment conditions were assessed in duplicate.

### 4.11. Data Analysis

FT-IR spectra were acquired and analyzed by the Spectrum IR software version 10.7.2 (PerkinElmer). UV-visible data were obtained using the Lambda XLS+ 8283 software version 1.1.0 (PerkinElmer). Absorbance values were reported to three decimal places and were not rounded. Wavenumber values were reported to two decimal places and were not rounded. HR-MS data analysis was performed using the Agilent MassHunter Qualitative Analysis software package version B.03.01 SP3. *m*/*z* values were reported for [M-H]^−^ ions. Statistical data analysis was performed with Microsoft Excel. Pairwise comparisons were assessed by a paired two-tailed Student’s *t*-test. A single-factor analysis of variance (ANOVA) was used when more than two test groups were compared with α = 0.05. Outlier detection was done using the Grubbs method with α = 0.01. Differences with a *p*-value smaller than 0.05 were considered statistically significant. All significant figures were retained throughout the analysis, and only the final value was rounded. Tabulated data were reported in the arithmetic mean ± standard deviation format. The formation constants for EDTA-metal ion complexes (log K_f_) used in the discussions are for the reaction M^n+^ + EDTA^4–^ ⇌ M(EDTA)^n–4^ at 25 °C and ionic strength 0.1 M [37].

## 5. Conclusions

Chelating agents are effective tools against heavy metal poisoning. However, the most used chelating agent, EDTA, has a major drawback, as it has previously been found to cause harmful side effects to humans. To overcome this obstacle, PEG-EDTA has been proposed as a potentially more biocompatible alternative to EDTA. In this study, we proposed an analytical approach to assess the utility of PEG-EDTA as a metal detoxification agent. Our data suggest that PEGylation of EDTA can make it more water-soluble and less cytotoxic; however, it also affects its ability to bind to metal ions due to chemical changes to the chelating agent, such as increased steric hindrance and loss of effective coordination sites.

Analytical methods, such as UV-visible spectrophotometry and displacement titration, were developed and used to comparatively assess metal ion complexation in EDTA and PEG-EDTA samples, using terbium(III) and zirconium(IV) ions. It was found that while PEG-EDTA was a less efficient chelating agent than EDTA, it posed less short-term toxicity to the tested human cells. Furthermore, zinc(II)-complexed PEG-EDTA was shown to be the least cytotoxic reagent, potentially due to its metal ion displacement properties, i.e., by removing more strongly binding (higher log K_f_) toxic metal ions without scavenging less strongly binding (lower log K_f_) essential metal ions. This property makes pre-complexed PEG-EDTA a potentially healthier heavy metal detoxification agent for human cells than EDTA and uncomplexed PEG-EDTA.

Through qualitative testing, certain properties of PEG-EDTA were also determined. PEG-EDTA formed stable complexes with selected heavy metal ions within a wide range of pH. These complexes were relatively stable in saline solutions and under UV exposure, suggesting the utility of PEG-EDTA for clinical applications. However, whether the reagent is useful for environmental or industrial applications under harsher conditions, such as prolonged exposure to sunlight or extremely ionic matrices, is yet to be determined.

In conclusion, PEG-EDTA and its zinc(II) complex elicit a great potential to be used as less toxic, but effective, metal detoxification agents instead of the ones currently in use, such as EDTA. Moreover, having improved physicochemical properties, PEG-EDTA could be used for various other applications, such as an additive in food products and personal care items, as well as for analytical uses. Although PEG-EDTA and its zinc(II) complex have been shown to be a less toxic alternative to EDTA for the human cell types used in this study, further studies are needed to confirm that they are fully biocompatible and harmless to humans. For example, the biological assays used in this study are limited to two human cell lines and a microscopy-based viability method. Additional cytotoxicity testing and dose-response experiments performed on these and other cell types are necessary to reveal the long-term effects of PEG-EDTA and its zinc(II) complex on humans. Animal studies and clinical trials can also ensure that these compounds are a safe alternative treatment option. The methods developed in this study for the determination of chelation can also potentially be adapted and applied to other chelating compounds to determine their metal ion-binding ability. Future research would include repeating the methods of analysis for other polycarboxylic chelating agents, such as DTPA, and comparing their chelation ability to EDTA and PEG-EDTA.

## Figures and Tables

**Table 1:** Calculated chelation efficiencies of EDTA and PEG-EDTA with Tb(III) and Zr(IV) ions using complexation colorimetry and displacement titration methods. Values are reported as mean ± standard deviation (*n* = 3).

Chelation	Tb(III)		Zr(IV)	
Efficiency (%)	Colorimetry	Titrimetry	Colorimetry	Titrimetry
EDTA	61.1 ± 0.1%	79.3 ± 0.1%	86.6 ± 0.1%	90.5 ± 0.2%
PEG-EDTA	60.2 ± 0.1%	77.4 ± 0.3%	79.7 ± 0.0%	86.9 ± 0.9%

**Table 2:** Carbonyl (C=O) stretching frequencies (in cm^−1^) of the PEGylation (${\bar{\nu }}_{e}$) and chelation (${\bar{\nu }}_{c}$) sites in PEG-EDTA complexes with Tb(III) and Zr(IV) ions under various treatment conditions. M and R represent the metal ion and the propargyl-PEG_4_ moiety, respectively. Values for uncomplexed PEG-EDTA (no metal ion) and unmodified EDTA (trisodium salt) are added for reference. Values are reported as mean ± standard deviation (*n* = 3). Single-factor ANOVA *p*-values are reported for each treatment condition at α = 0.05.

	Carbonyl Stretching Frequency, cm^−1^			
Treatment	Tb(III)		Zr(IV)	
	${\bar{\nu }}_{e}$ RO–(C=O)–	${\bar{\nu }}_{c}$ –(C=O)–OM	${\bar{\nu }}_{e}$ RO–(C=O)–	${\bar{\nu }}_{c}$ –(C=O)–OM
pH 2.2	1732.67 ± 1.85	1608.09 ± 2.24	1745.84 ± 0.16	1646.91 ± 0.45
pH 6.5	1737.32 ± 0.28	1605.70 ± 0.13	1743.99 ± 0.12	1646.06 ± 0.78
pH 8.5	1737.06 ± 0.01	1594.65 ± 0.34	1737.96 ± 0.12	1648.91 ± 2.46
pH 10.0	1741.58 ± 0.08	1590.96 ± 0.04	1735.95 ± 0.18	1647.10 ± 1.39
*p* acidity	2.29 × 10^−5^	1.69 × 10^−7^	1.35 × 10^−12^	1.97 × 10^−1^
0.010 M NaCl	1739.72 ± 0.62	1603.07 ± 0.03	1739.93 ± 0.13	1648.58 ± 1.80
0.10 M NaCl	1740.80 ± 0.27	1614.00 ± 0.00	1743.75 ± 0.13	1648.56 ± 1.22
1.0 M NaCl	1741.53 ± 0.47	1617.37 ± 0.05	1747.79 ± 0.12	1650.75 ± 1.68
*p* salinity	9.72 × 10^−3^	6.76 × 10^−15^	1.10 × 10^−9^	2.32 × 10^−1^
4.0 °C	1742.91 ± 0.18	1606.11 ± 0.08	1741.83 ± 3.15	1646.00 ± 1.03
60.0 °C	1742.76 ± 0.19	1610.57 ± 0.40	1740.80 ± 1.59	1649.08 ± 3.00
90.0 °C	1738.11 ± 0.33	1612.25 ± 0.08	1737.67 ± 1.47	1650.14 ± 1.61
*p* temperature	4.68 × 10^−7^	1.90 × 10^−7^	1.32 × 10^−1^	1.09 × 10^−1^
0.5 min UV	1741.81 ± 0.14	1601.73 ± 2.06	1741.81 ± 0.13	1645.57 ± 1.01
1.0 min UV	1741.58 ± 0.17	1607.12 ± 0.05	1741.72 ± 0.09	1645.51 ± 1.35
5.0 min UV	1741.63 ± 0.16	1612.83 ± 1.72	1745.90 ± 0.18	1646.13 ± 1.31
*p* UV exposure	2.52 × 10^−1^	3.75 × 10^−4^	3.91 × 10^−8^	8.02 × 10^−1^
Uncomplexed PEG-EDTA	1731.00 ± 0.21	1666.00 ± 0.16	1731.00 ± 0.21	1666.00 ± 0.16
Unmodified EDTA	N/A	1668.59 ± 0.06	N/A	1668.59 ± 0.06

**Table 3:** Viability of normal/healthy (HUVEC) and cancerous (TNBC) human cells treated with a cytotoxic heavy metal ion with or without EDTA or PEG-EDTA. Values are reported as mean ± standard deviation (*n* = 3 and *n* = 2 for HUVECs and TNBC cells, respectively).

Sample	Cell Type	Metal Ion	Chelating Agent	Displaced Metal Ion	Cell Viability (%)
**A**	HUVEC	-	-	-	95.6 ± 2.1
**B**	HUVEC	Pb(II)	-	-	42.3 ± 3.6
**C**	HUVEC	Pb(II)	EDTA	-	77.4 ± 7.7
**D**	HUVEC	Pb(II)	PEG-EDTA	-	71.0 ± 12.1
**E**	HUVEC	Pb(II)	EDTA	Zn(II)	83.1 ± 5.8
**F**	HUVEC	Pb(II)	PEG-EDTA	Zn(II)	89.6 ± 4.2
**G**	TNBC	-	-	-	95.9 ± 0.6
**H**	TNBC	Ag(I)	-	-	13.5 ± 0.3
**I**	TNBC	Ag(I)	EDTA	-	78.2 ± 0.2
**J**	TNBC	Ag(I)	PEG-EDTA	-	94.4 ± 2.8
**K**	TNBC	-	EDTA	-	92.6 ± 6.2
**L**	TNBC	-	PEG-EDTA	-	92.4 ± 2.9

**Table 4:** Chemical reactions for colorimetric and titrimetric quantitation of free (uncomplexed) Tb(III) and Zr(IV) ions in solution. Reactions **A** and **B** use CAS as the colorimetric reagent. Reactions **C** and **D** rely on displacing Ni(II) with the analyte, then titrating the liberated Ni(II) with standardized EDTA in the presence of Cu-PAN as the titration endpoint indicator. Note that the EDTA added in the last step of reactions **C** and **D** is the titrant. The experiments were performed at pH 4.2, and in triplicate.

	**Chemical Reactions**
**A**	${Tb}^{3+}+{CAS}^{3-}\to Tb{\left(CAS\right)}^{0}$ →($\lambda_{max}=500\;nm$)
**B**	${Zr}^{4+}+{CAS}^{3-}\to Zr{\left(CAS\right)}^{+}$ →($\lambda_{max}=598\;nm$)
**C**	$2{Tb}^{3+}+3Ni{\left(CN\right)}_{4}^{2-}\to 2Tb{\left(CN\right)}_{6}^{3-}+{3Ni}^{2+}$ ${3Ni}^{2+}+{3H_{2}(EDTA)}^{2-}\to 6H^{+}+3Ni{\left(EDTA\right)}^{2-}$(pale yellow)
**D**	${Zr}^{4+}+2Ni{\left(CN\right)}_{4}^{2-}\to Zr{\left(CN\right)}_{8}^{4-}+{2Ni}^{2+}$ ${2Ni}^{2+}+{2H_{2}(EDTA)}^{2-}\to 4H^{+}+2Ni{\left(EDTA\right)}^{2-}$(pale yellow)

## Data Availability

See Supplementary Materials for PEG-EDTA characterization data.

## Supplementary Materials

The following supporting information can be downloaded at: https://www.mdpi.com/article/10.3390/ijms27010044/s1.

## Abbreviations

The following abbreviations are used in this manuscript:

ATR	attenuated total reflectance
CAS	chrome azurol S
Cu-PAN	copper 1-(2-pyridylazo)-2-naphthol
DTPA	diethylenetriaminepentaacetic acid
ECGS	endothelial cell growth supplement
EDTA	ethylenediaminetetraacetic acid
ESI-oaTOF	electrospray ionization-orthogonal acceleration time-of-flight
FBS	fetal bovine serum
FT-IR	Fourier transform-infrared
HR-MS	high-resolution mass spectrometry
HUVECs	human umbilical vein endothelial cells
ICP-MS	inductively coupled plasma mass spectrometry
PEG	polyethylene glycol
TNBC	triple-negative breast cancer
UV	ultraviolet
