# Supplementary material for: Protective Effect of PEG-EDTA and Its Zinc(II) Complex on Human Cells
Source: Int J Mol Sci. 2025 Dec 20;27(1):44. doi: 10.3390/ijms27010044 (PMC12785647; doi:10.3390/ijms27010044)
Supplement: Supplementary file 1 [file ijms-27-00044-s001.zip › ijms-4000044-supplementary.pdf]

Protective effect of PEG-EDTA and its zinc(II) complex on human cells

Tashneet Dhaliwal, Cole Babcock, Brynmar Degenhardt, Isaac Osorio Passos, Tigran Stepanyan and Makan Golizeh

SUPPLEMENTAL INFORMATION

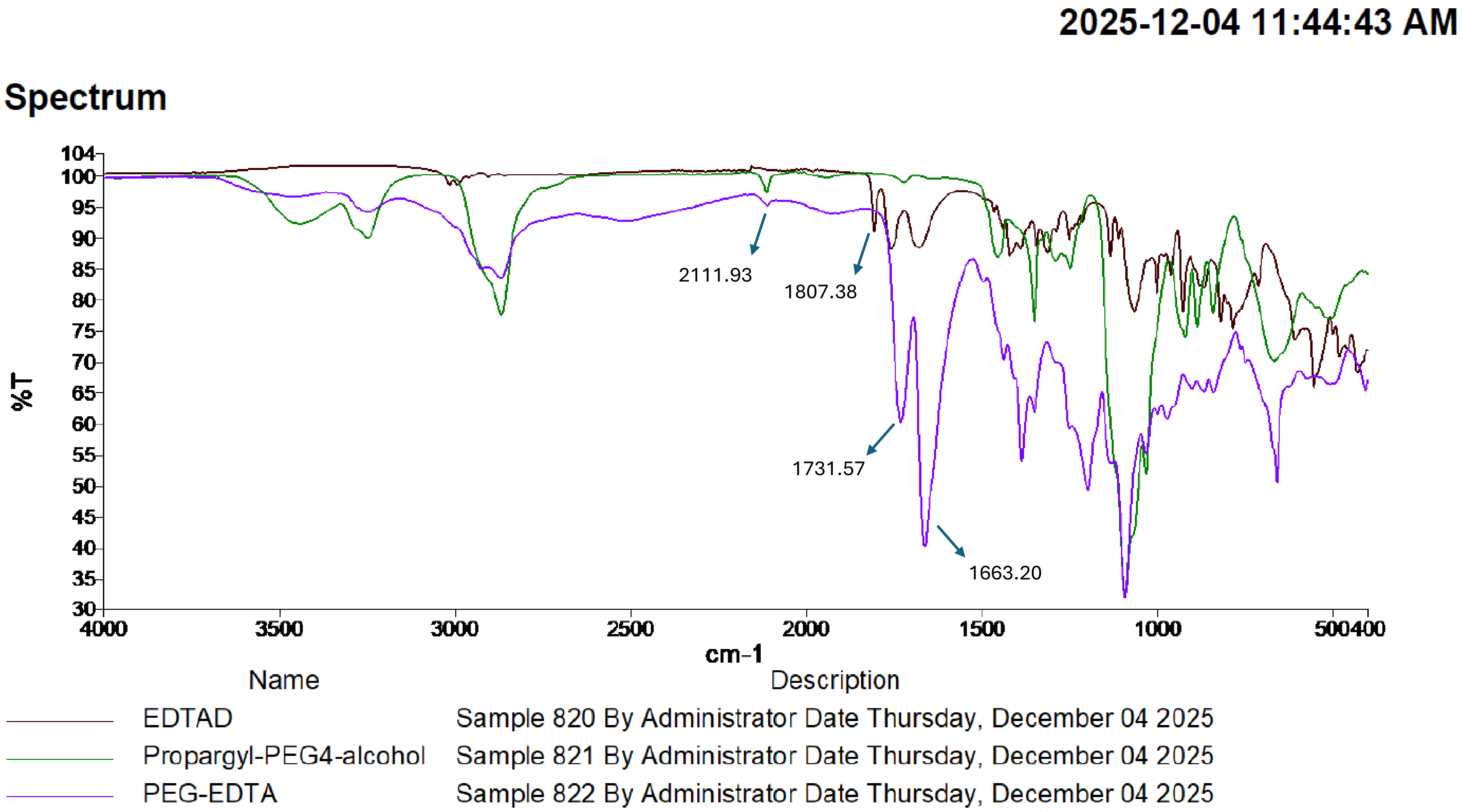

Figure S1. Fourier transform-infrared (FT-IR) spectrum of the synthesized PEG-EDTA product and its reactants. The disappearance of the anhydride carbonyl stretching peak at 1807.38 cm^{-1} confirms the removal of EDTA dianhydride (EDTAD) after reaction workup. The high-intensity peaks at 1663.20 cm^{-1} and 1731.57 cm^{-1} suggest the formation of the free carboxylates of PEG-EDTA. The peak at 2111.93 cm^{-1} confirms the incorporation of the propargyl moiety into the product.

Qualitative Analysis Report

**Data Filename**	25120223.d	**Name**	C. Babcock, Concordia U.
**Sample Name**	peg-edta	**Position**	-1
**Instrument Name**	oaTOF6220	**Operator**	ami
**Acq Method**		**DA Method**	ami_da.m

User Spectra

Fragmentor Voltage	Collision Energy	Ionization Mode
100	0	Esi

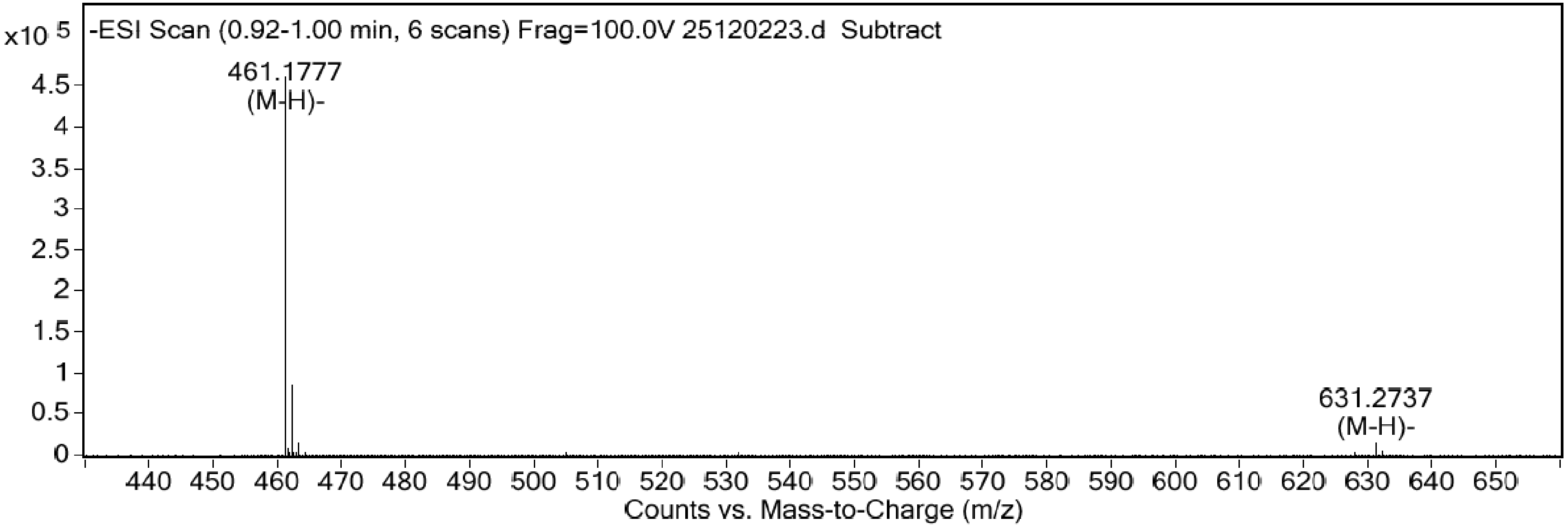

Formula Calculator Results

Formula	Ion Formula	Mass	Calc Mass	m/z	Calc. m/z	Diff (mDa)	Diff (ppm)	DBE	Ion Species	Score
C19 H30 N2 O11	C19 H29 N2 O11	462.1849	462.185	461.1777	461.1777	0.02	0.05	6	(M-H)-	93.33
C28 H44 N2 O14	C28 H43 N2 O14	632.281	632.2793	631.2737	631.272	-1.77	-2.8	8	(M-H)-	93.49

--- End Of Report ---

Figure S2. Electrospray ionization-orthogonal acceleration time-of-flight (ESI-oaTOF) mass spectrum of the synthesized PEG-EDTA product. The $[M-H]^-$ ion at *m/z* 461.1777 is associated with deprotonated monosubstituted PEG-EDTA with a mass accuracy of 0.05 ppm. The $[M-H]^-$ ion at *m/z* 631.2737 is associated with small amounts of deprotonated disubstituted $(PEG)_2$EDTA with a mass accuracy of -2.8 ppm. No other peaks has been detected in the product.
